# Procedures for obtaining muscle physiology parameters during a gracilis free-functioning muscle transfer in adult patients with brachial plexus injury

**DOI:** 10.1038/s41598-022-09861-y

**Published:** 2022-04-12

**Authors:** Lomas S. Persad, Filiz Ates, Loribeth Q. Evertz, William J. Litchy, Richard L. Lieber, Kenton R. Kaufman, Alexander Y. Shin

**Affiliations:** 1 Department of Orthopedic Surgery, Mayo Clinic, Rochester, MN USA; 2 Department of Neurology, Mayo Clinic, Rochester, MN USA; 3 Shirley Ryan Ability Lab, (Rehabilitation Institute of Chicago), Chicago, IL USA; 4 Hines V.A. Hospital, Maywood, IL USA; 5 Northwestern University, Chicago, IL USA; 6 Aerospace Engineering and Geodesy, University of Stuttgart, Stuttgart, Germany; 7 Montana State University, Bozeman, USA; 8 Motion Analysis Laboratory, 200 First Street SW, DAHLC 4-214, Rochester, MN 55905 USA

**Keywords:** Skeletal muscle, Physiology

## Abstract

A complete understanding of muscle mechanics allows for the creation of models that closely mimic human muscle function so they can be used to study human locomotion and evaluate surgical intervention. This includes knowledge of muscle–tendon parameters required for accurate prediction of muscle forces. However, few studies report experimental data obtained directly from whole human muscle due to the invasive nature of these experiments. This article presents an intraoperative, in vivo measurement protocol for whole muscle–tendon parameters that include muscle–tendon unit length, sarcomere length, passive tension, and active tension in response to external stimulation. The advantage of this protocol is the ability to obtain these rare experimental data in situ in addition to muscle volume and weight since the gracilis is also completely removed from the leg. The entire protocol including the surgical steps for gracilis harvest takes ~ 3 h. Actual testing of the gracilis where experimental data is measured takes place within a 30-min window during surgery.

## Introduction

Quantifying human muscle mechanical properties is an important step in understanding in vivo muscle function as well as developing computational muscle models. Musculoskeletal models are used in the study of human movement and the effects of surgical intervention but almost never developed using data directly obtained from humans. To ensure physiological accuracy, predictions made by such models must be validated against actual experimental data. However, it is extremely difficult to obtain physiological data from living human muscle. Thus, musculoskeletal models typically rely on inferred data from experiments performed on cadavers or isolated, living muscle in animals^1,2^. Obtaining experimental data from living humans remains a challenge. In response to this challenge, this protocol outlines the process of quantifying gracilis mechanical properties in patients who are undergoing a gracilis free functional muscle transfer surgery. The surgery is performed to restore upper extremity function in patients who had a traumatic brachial plexus injury. In this protocol, we describe measurement of in situ force–length properties at the time of surgery. We also provide details as to how the methods of muscle biopsy using a custom biopsy clamp can be used to measure sarcomere length via laser diffraction^3^.

### Development of the protocol

Adult brachial plexus injuries (BPI) occur due to trauma and are most seen in motor vehicle accidents^4^. The mechanism of injury is violent or sudden extreme rotation of the head or shoulder which stretches or ruptures the nerves resulting in a BPI. Adult traumatic BPI are increasing due to the growing popularity of extreme sports and rising number of vehicular accident survivors^5–7^. Most patients require surgery to restore elbow flexion. There is a time dependant degeneration of motor end plates following injury. Surgical intervention, when possible, should occur within 6–10 months after injury to avoid this degradation^8^. When feasible, nerve grafting of viable nerve roots or nerve transfers are performed. In late cases or when restoration of rudimentary grasp is desired, a unique surgical procedure exists where a leg muscle (gracilis) is surgically harvested from the patient’s leg and transplanted into their arm to restore elbow flexion^9^. Since the gracilis is fully exposed during the muscle transfer, it represents an unprecedented opportunity to gather muscle physiological properties. The gracilis muscle function is characterized by measuring key parameters of sarcomere length, muscle–tendon length, muscle mass, and passive and active force–length properties. Pilot studies were performed to test the feasibility and logistics of measuring these parameters and these studies were used to develop the current protocol.

All methods used in this research involve experiments with human participants. This research study is performed in accordance with the Declaration of Helsinki. Informed consent is obtained in accordance with relevant guidelines and regulations for human subject research as specified in 45 CFR 46.116-General Requirements for Informed Consent and 21 CFR 50.25-Elements of Informed Consent. Each research subject provides their informed written consent for study participation. Each subject agrees to the use of their image, if needed, for publication purposes. The protocol is approved by the Institutional Review Board of Mayo Clinic. We successfully demonstrated a viable, reproducible protocol to obtain in vivo normal human gracilis muscle–tendon length, sarcomere length, muscle architecture, high-fidelity active and passive force data.

### Validation studies

Data obtained using this protocol were used to test the validity of predictions from a commonly used musculoskeletal models in OpenSim. Our results show that there are significant errors in muscle length predicted from current musculoskeletal models^10^. The greatest error occurred when the knee was fully extended with an absolute average error of muscle–tendon length of 7% and absolute average fiber length error between 15 and 32%. Furthermore, it was observed that the manual linear scaling method that was used by the models developed using OpenSim^11^ did not capture the normal variability in muscle–tendon length observed in human subjects.

Additionally, we showed that the human gracilis muscle has passive mechanical properties that are more like rodent muscle than rabbit muscle since it was observed there was only a modest increase in gracilis passive modulus when going from single fiber to whole muscle. One key finding from this study was that sarcomere strain rather than muscle strain is the best predictor of passive skeletal muscle stress^12^.

### Applications of the method

The methodology to obtain active and passive mechanical properties of human skeletal muscle may be applied to other muscles for free functional muscle transfer or surgical procedures where the muscle and associated nerve, artery, and vein are exposed.

### Comparison with other methods

Elucidating muscle function in relation to the joints it crosses is crucial for understanding human locomotion, however, direct measurements of individual human muscle force is very rare. In a few studies, force was measured from human Achilles tendon^13^. The data on triceps surae muscle group^14^ provided important but limited information during walking. Initially, force–length characteristics of upper extremity muscles were obtained from amputees^15^. The approach was modified, improved, and used on muscles of patients with cerebral palsy during tendon transfer surgeries^16–18^. These earlier intraoperative data collections on upper extremity muscles provided limited data. Data collection was performed after the tendon was divided and measurement of muscle force production capacity at specific joint positions was not possible. Recent intraoperative studies focused on collecting isometric muscle force with respect to joint angle position^19,20^. Implementation of buckle force transducers (BFTs) to human tendons enabled to measure muscle forces from an intact tendon. Using this approach, force–length characteristics of healthy gracilis muscle were reported e.g., during anterior cruciate ligament surgery^21^ and hamstring muscles of patients with cerebral palsy who undergo muscle lengthening surgeries^22,23^.

The main advantages of the present protocol are that (1) it allows us to measure the dimensions, shape, and weight of the whole muscle since the gracilis is completely removed from its native location. These can only be obtained during free muscle transfer surgeries. (2) This protocol is unique in that it provides an opportunity to relate not only the muscle force with the joint position but also with the sarcomere length.

### Experimental design

Due to the unique and complex nature of brachial plexus reconstructive surgery involving a gracilis free functional muscle transfer, ideally the procedures are performed by two teams of surgeons to reduce anesthesia and surgical time. In this situation, one team of surgeons prepare brachial plexus nerves to be grafted or transferred as well as the donor nerve(s) and vessels for the gracilis transfer, while the second team harvests the myocutaneous free functioning gracilis. When done in this manner, there is a short window of time that the gracilis harvest team has before the upper extremity team is ready to receive the gracilis muscle. During this 20–30 min interval, muscle force–length data is obtained and does not disrupt the progress of the surgeons on the upper extremity or delay surgical progress.

### Expertise needed to implement the protocol

Personnel required to execute the protocol include:

- *Orthopedic hand surgeon and surgical assistant*: The orthopedic hand surgeon with expertise in microvascular surgery commences harvest of the gracilis muscle prior to testing, mounts the force transducer on the tendon and obtains the biopsy samples. The surgical assistant aids the surgeon in stabilizing the leg during muscle stimulation, measuring muscle–tendon unit (MTU) length. This team is part of the brachial plexus reconstruction team.
- *Neurophysiologist*: They are responsible for setup of the intraoperative electromyography system and performing the muscle stimulation trials. This team is otherwise a normal part of the surgical reconstruction for intraoperative nerve testing of the brachial plexus roots.
- *Research scientist*: They are responsible for data acquisition system set up that connects to the BFT, collects intraoperative data, and secures biopsy samples. They must be familiar with digital data acquisition systems, understand signal conditioning concepts, electronic instrumentation and can perform maintenance and calibration of the BFT.
- *Photographer*: They are responsible for recording a video of the muscle for volumetric analysis as well as capturing images of the harvested gracilis when placed on a sterile towel and adjacent to a sterile ruler. Muscle and tendon length are measured from these images. They are called only when needed and are not present for the duration of the study.

**Figure 1:**
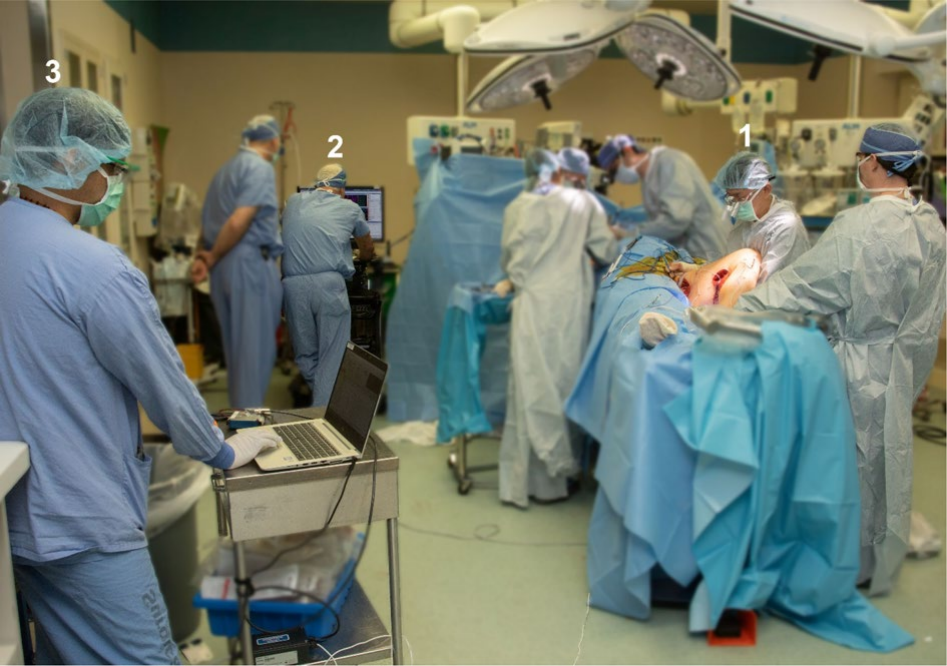
Layout of operating room for intraoperative muscle force measurement. (1) The surgeon and assistant hold the leg in position while the surgeon keeps the stimulator probe on the obturator nerve during stimulation. (2) Neurophysiologist determines the settings for maximum compound muscle action potential (CMAP) then performs the 2 Hz and 20 Hz stimulation at 50% and 25% of the current required to produce maximal CMAP. (3) Research scientist records the output from the BFT using a custom LabVIEW interface. The neurophysiologist coordinates with the research scientist to indicate the start of each stimulation trial.

Figure 1 shows the layout of the personnel in the operating room so they can all work together without interfering with the overall surgical procedure. The research scientist and neurophysiologist are on standby until the surgeon gives the indication that the experimental protocol can be performed.

### Limitations

The limitations that must be considered when using this protocol include:

- MTU length, force measurements and biopsy samples are obtained at multiple joint configurations. Therefore, the protocol requires repeated positioning of the leg at different joint configurations with predetermined joint angles. This source of error can be limited by having a single surgeon (AYS) position the leg for all subjects and choosing joint angles that can be easily approximated such as 45°, 60°, or 90° (refer to Fig. 7).
- There will be minor unwanted leg movement during muscle stimulation since the patient’s leg is being held at the required joint configuration as the muscle contracts. This source of error is reduced by stimulating the muscle submaximally and thereby reducing muscle force production to manageable levels.
- The submaximal muscle activation results in a reduced contraction and as such may not correctly represent the true force-generating potential of the gracilis. This has implications when modeling in vivo muscle function since the Hill-type models that are used are more accurate at predicting muscle forces under maximally activated conditions^24^. This issue can be addressed by calculating the level of muscle activation then scaling the peak force values to maximal activation conditions. First, calculate the ratio of peak force for the 2 Hz twitch at submaximal activation to peak force of the CMAP twitch at 100% activation. This ratio estimates muscle activation during the stimulation trials. We found that this ratio was close to 0.8 therefore it was appropriate to linearly increase the experimental peak force to estimate maximal activation peak force.
- Active force measurement using the BFT prevents the use of electrocautery during the concurrent surgery at the upper extremity recipient site. The BFT signal is sensitive to this electrical interference resulting in a noisy signal. Careful cooperation and communication with the upper extremity team is necessary to limit delays during crucial stages of the surgery.

## Materials

### Human subjects

#### Caution

All research protocols must be approved by an Institutional Review Board. Subjects must be over 18 years old and scheduled to undergo a gracilis free functioning muscle transfer surgery to restore elbow flexion as part of their brachial plexus injury reconstruction^9,25^. Subjects must give written and informed consent to undergo the intraoperative gracilis muscle data collection as part of their brachial plexus reconstruction.

### Equipment

#### Critical

These items are required for the experimental protocol. They are categorized according to equipment handled by the surgeon (and therefore must be sterile) and those handled by the research scientist (located on the periphery of the surgical field and nonsterile) collecting the intraoperative data. The biopsy clamps are steam sterilized and the BFT is ethylene oxide (ETO) sterilized per the institutional sterility requirements. The data acquisition software and hardware listed below are examples. It is not necessary to use these specific products for this protocol.

##### Sterile equipment for surgeon

**Figure 2:**
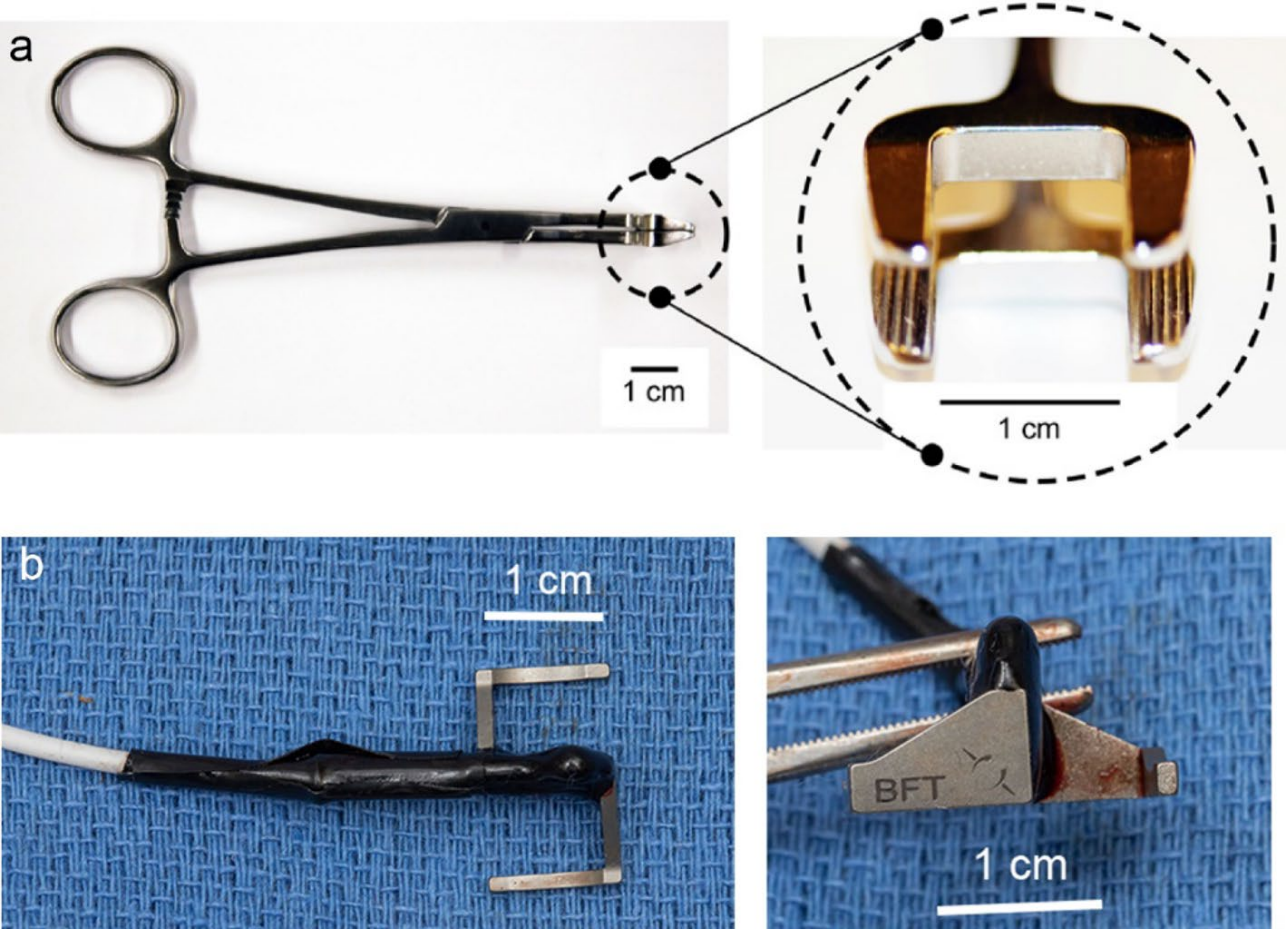
Biopsy clamp and BFT. (**a**) Side view of modified biopsy clamp and close up view of clamp jaws. (**b**) Top and side view of BFT.

- Biopsy clamp^26^ in Fig. 2a (14 cm modified hemostat clamp, Sabri Group, Pompano Beach, FL, USA model 3-113-14)
- BFT in Fig. 2b (× 2) (S-shape, dimensions: width = 10 mm length = 20 mm and height = 9 mm, TEKNOFIL, Istanbul, Turkey)
- Plastic bowl (Medline DYND50315, Medline Industries, Mundelein, IL)
- Ruler (Medline DYNJRULER, Medline Industries, Mundelein, IL)
- #2 FiberWire suture with needle removed (AR-7200, Arthrex, Naples, FL)
- Titanium Liga-Clips (Ethicon LigaClip MCA Medium, Johnson and Johnson, Cincinnati OH)
- Nerve stimulator probe (90 mm Cadwell disposable side-by-side bipolar probe, Cadwell, Kennewick, WA, cat. no. 302429-00)
- Subdermal needle electrodes (Cadwell twisted disposable subdermal needle electrode, Cadwell, Kennewick, WA, cat. no. 302821-000-060)

##### Nonsterile research equipment

- Laptop with LabView (2019 version, National Instruments, Austin, TX)
- Data acquisition system (USB 6000, National Instruments, Austin, TX)
- Strain gauge amplifier (Model SGA, Interface force measurement solutions, Scottsdale, AZ)
- Intraoperative Neuromonitoring Instrument (Cadwell Cascade Pro, Cadwell, Kennewick, WA)
- 200 mL storage bottles (Corning 431431, Corning Incorporated, Corning, NY)
- 50 mL conical tubes (Corning 352098, Corning Incorporated, Corning, NY)
- Parafilm (Parafilm “M”, American Can Co., Greenwich, CT)
- Digital scale (CS-5000, Ohaus Corp., Parsippany, NJ)
- Digital camera (EOS 5d mark iv, Canon U.S.A. Inc., MELVILLE, NY)
- Floor cord cover (optional)

##### Reagents

- 10 × phosphate-buffered saline (PBS)
- 10% buffered Formalin

##### Reagent setup

**1X PBS solution**. Dissolve 20 mL of 10X PBS in 180 mL distilled water.

### Buckle force transducer (BFT) calibration

**Figure 3:**
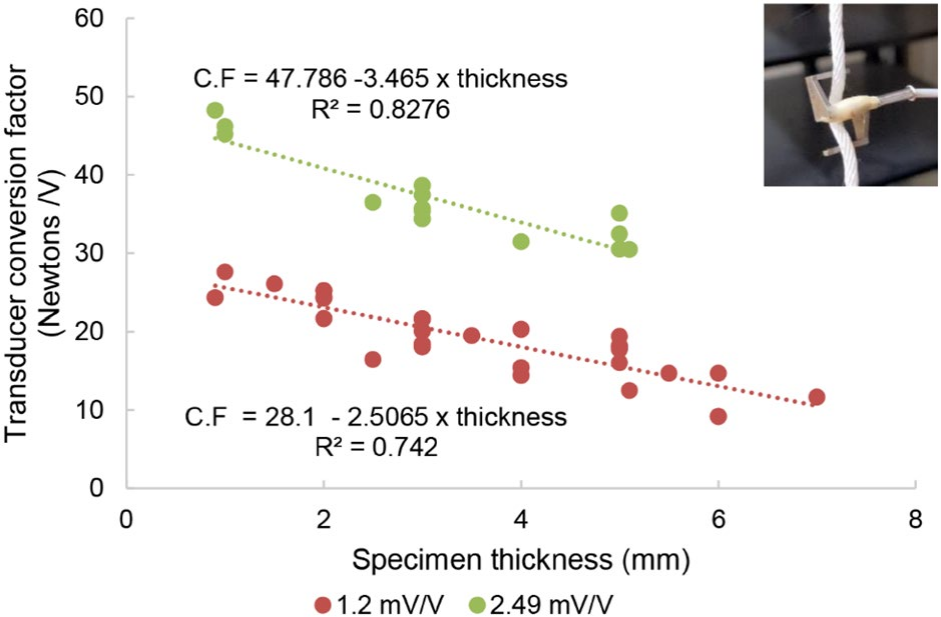
Conversion factor (C.F) for the BFT as a function of specimen thickness. Data is shown for two gain settings (1.2 mV/V and 2.49 mV/V). Inset image shows an example of material used to calibrate the force transducer in the laboratory.

The BFT is calibrated in the laboratory according to methods described by An et al.^27^. First, the BFT and data acquisition system are connected. The voltage offset on the strain gauge amplifier (Model SGA, Interface force measurement solutions, Scottsdale, AZ) is adjusted until the output of the BFT is zero, meaning that the Wheatstone bridge circuit of the BFT is balanced. The BFT is mounted on a length of rope (inset image Fig. 3) and voltage is recorded as four 0.9 kg (two-pound) weights are incrementally suspended to calibrate the BFT between 0 and 36 N. The calibration curve of force *versus* voltage is plotted resulting in a linear relationship with a coefficient of determination exceeding 0.9. The slope of this relationship yields the conversion factor (in units of NV^−1^) for that specific BFT. To account for subject specific tendon thickness, regression analysis is used to correct the conversion factor as a function of tendon thickness. Therefore, the calibration steps are repeated for materials of varying thickness (1–7 mm) (Fig. 3).

### Custom data acquisition program

**Figure 4:**
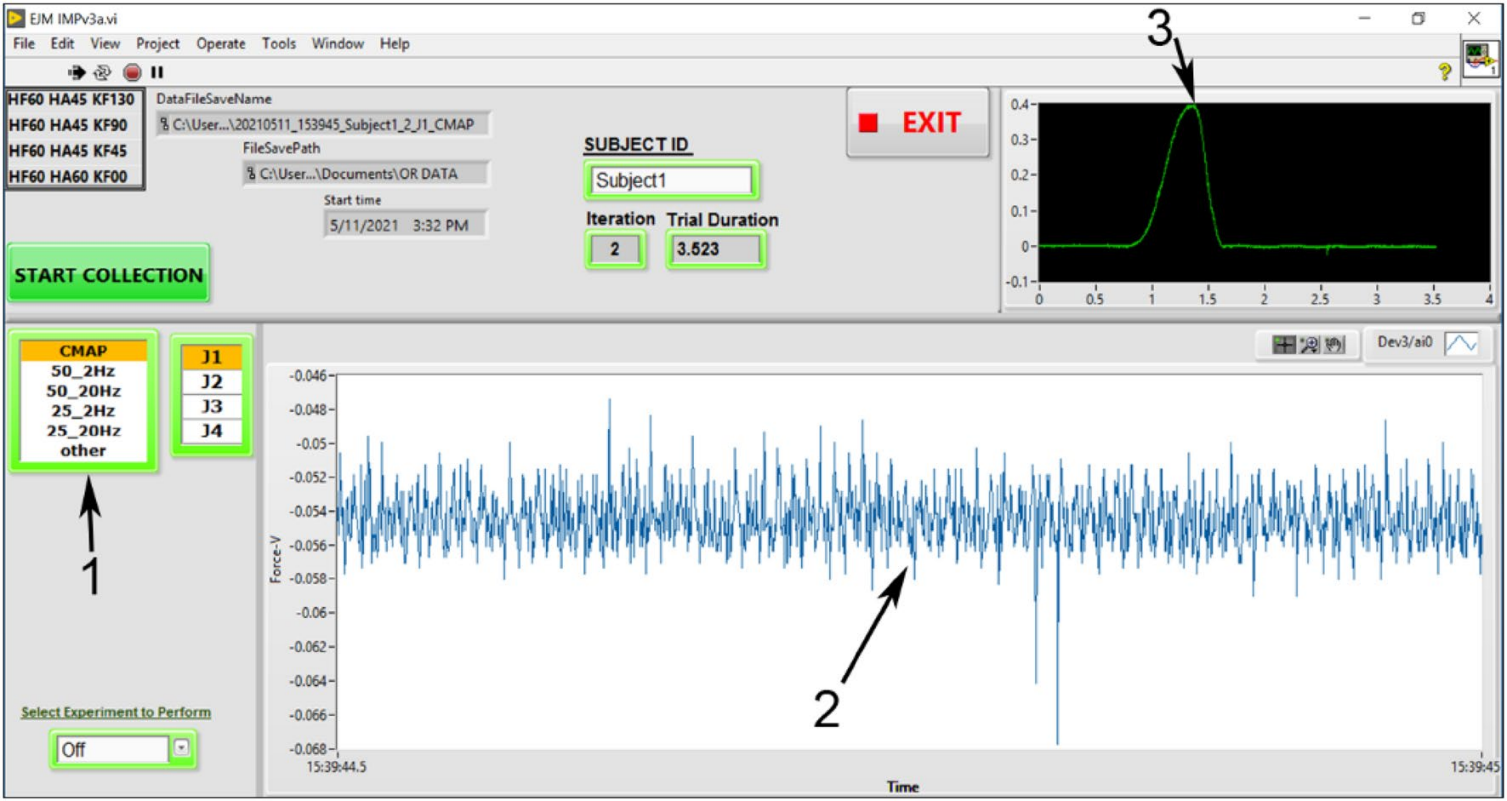
LabVIEW VI user interface. (1) Trial type selection. (2) Live feed from BFT. (3) Previously captured trial preview.

The amplified signal from the BFT is collected with a data acquisition system (USB-6000, National Instruments) at a sampling frequency of 1 kHz. For the custom user interface, we recommend that the display show the live feed from the BFT to the operating room staff so that they can monitor its function as well as a separate display showing the previous trial that was collected. The custom LabVIEW user interface is shown in Fig. 4 for reference.

## Phases of the procedure

The procedure is broken into phases listed below. These phases are also identified within the procedure section where the experimental instructions are listed.

### Setup of USB data acquisition device (DAQ)

**Figure 5:**
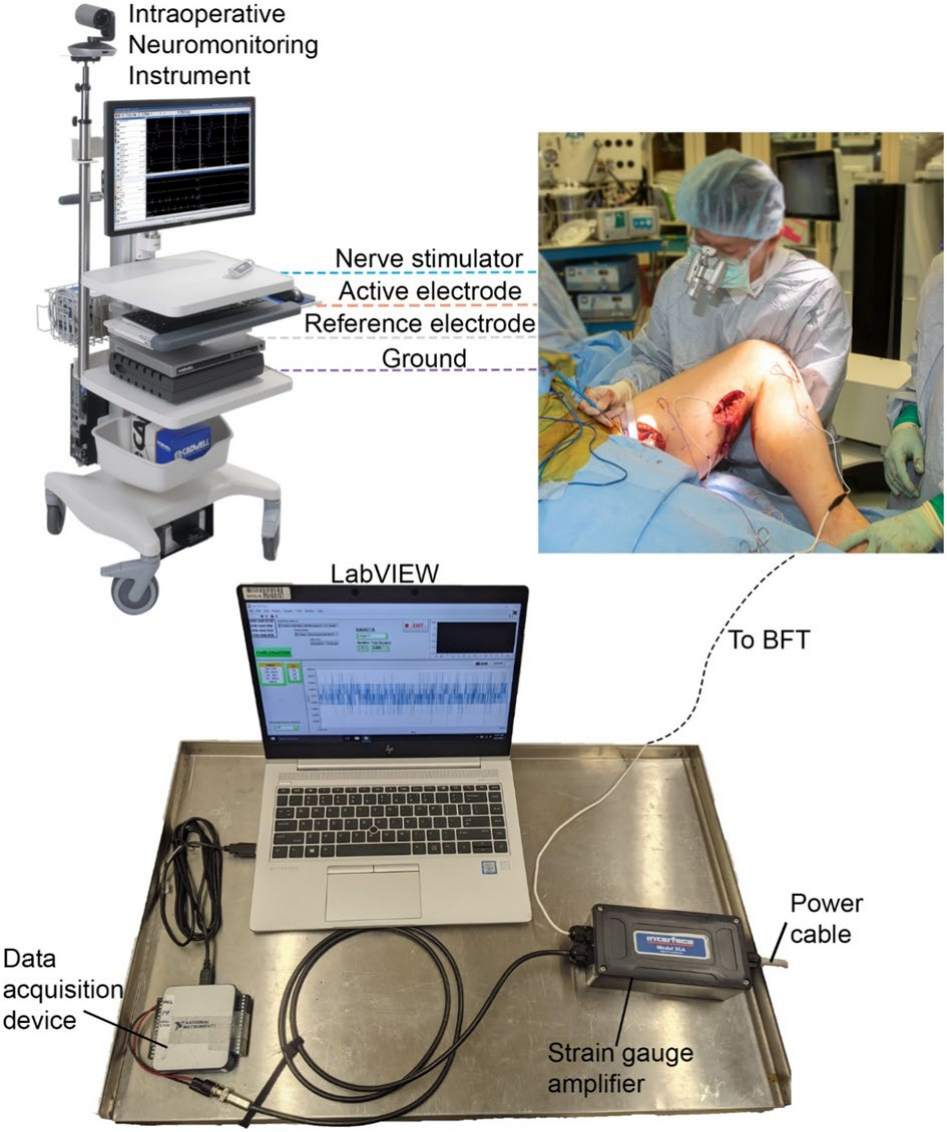
Experimental setup. Foreground shows the devices that are used to record the BFT signal. The BFT cable from the patient is connected to the strain gauge amplifier input. The strain gauge amplifier output is connected to the DAQ which is connected to a laptop via a USB connection. Data collection is performed using LabVIEW. The nerve stimulator and electrodes are connected to an intraoperative neuromonitoring instrument (Cadwell Cascade Pro, Cadwell, Kennewick, WA). Details of EMG electrode placement are described in step 16 and Fig. 10.

Calibration is performed in the laboratory prior to the surgery. Therefore, all devices should just be connected, powered on and ready to go. A schematic of the BFT setup is shown in foreground of Fig. 5. The research scientist performs this setup while the surgeon prepares the gracilis to be harvested.

### Surgical technique for preparing the gracilis MTU

**Figure 6:**
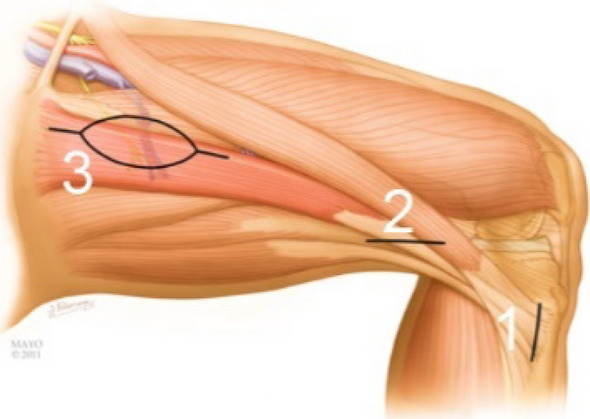
Three incisions made to harvest the gracilis. (1) Longitudinal incision made over the *pes anserine*. (2) Distal medial thigh incision at a length of the musculotendon junction. (3) Proximal medial thigh incision. (Printed with permission of the Mayo Foundation for Medical Education and Research. All rights reserved, copyright 2010).

The steps for harvesting the gracilis are modified from the description by Giuffre et al*.*^25^, leaving the muscle origin and insertion intact until testing is completed. The contralateral gracilis is typically used as the donor muscle secondary to the vascular pedicle orientation to the recipient vessels of the side of the brachial plexus injury. Exposure of the gracilis occurs simultaneously with the operative team exposing the brachial plexus, the infraclavicular vessels and recipient site. Three incisions made to harvest the gracilis (Fig. 6).

Short term paralytic agents are used for induction of anesthesia. After induction, no inhalational anesthetics are used during the exploration of the brachial plexus, harvest and testing of the muscle. Total Intravenous Anesthesia (TIVA) is utilized to optimize intraoperative nerve testing of the brachial plexus. Once evoked potentials are obtained, anesthesia is switched to inhalational anesthetics. TIVA is used during the entire muscle harvest and testing. The most commonly used TIVA agent is Propofol augmented with an opioid.

### Intraoperative measurement

**Figure 7:**
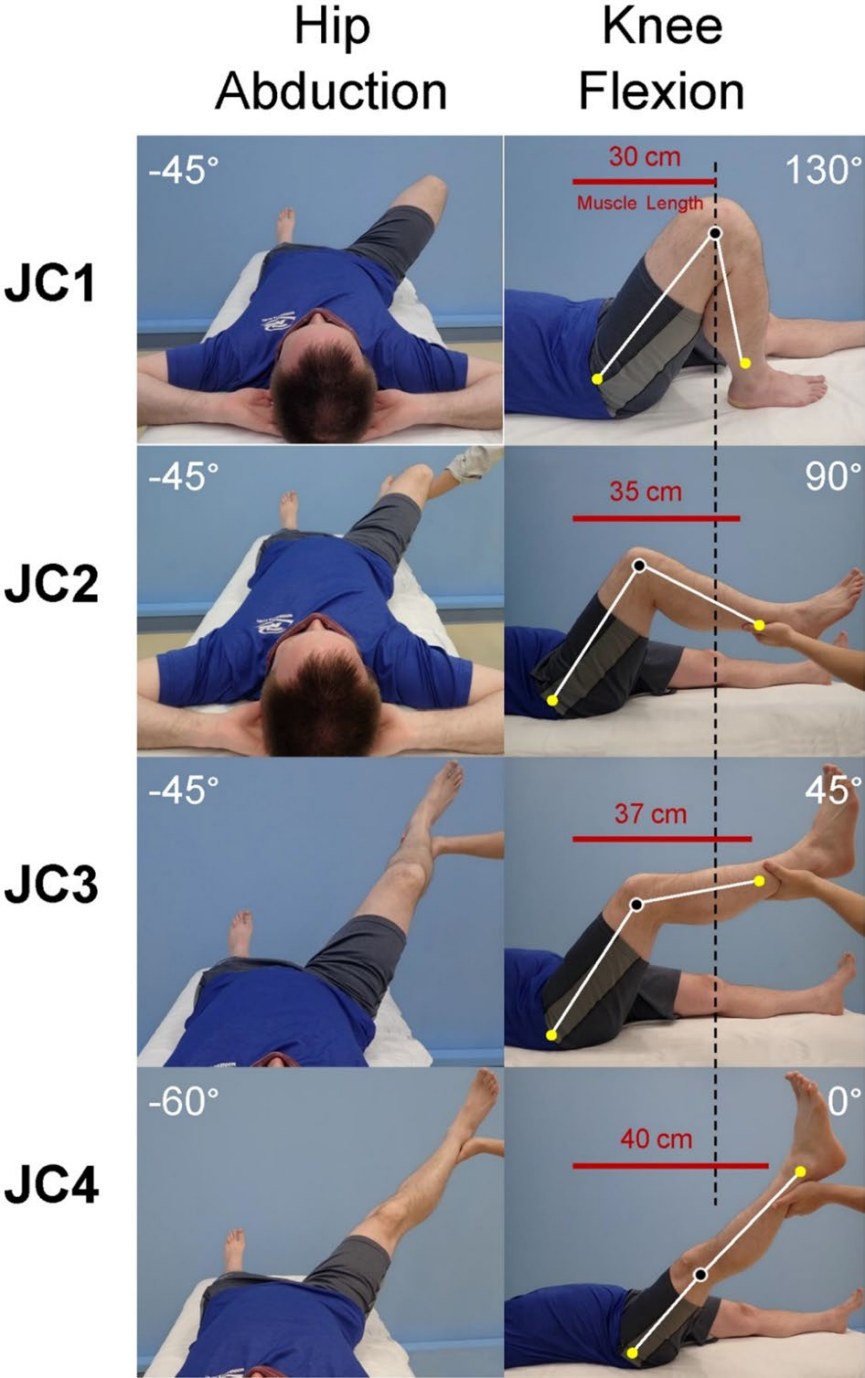
Simulation of the four intraoperative joint configuration (JC) used during data collection. The first column shows hip abduction and the second column shows knee flexion. Hip flexion was 60°. Muscle length increases from 30 to 40 cm from JC1 to JC4.

Force and length are measured from the MTU in situ with the muscle origin and insertion intact. These data are recorded at four joint configurations (Fig. 7). As the limb is placed in the various joint configuration the MTU is lengthened.

### Measurement of MTU length

MTU length is measured using suture held at the origin and insertion of the gracilis and marked using surgical clips.

### Measurement of muscle force with intraoperative stimulation of the motor nerve

#### Critical

In vivo muscle force is measured by stimulating the muscle via the obturator nerve and measuring the muscle passive and active forces using a BFT mounted on the gracilis distal tendon. The surgeon first mounts and secures the BFT on the gracilis tendon then inserts the EMG electrodes required for stimulating the muscle. The obturator nerve is first stimulated to determine the current required to produce a maximal compound muscle action potential (CMAP). For data acquisition this current is reduced by 50% and 25% to better stabilize the leg and prevent excessive forces from being generated thereby ensuring isometric conditions. We recommend having a backup sterilized BFT ready to be quickly substituted if any technical problems arise.

### Muscle biopsy

Muscle tissue samples are taken from the exposed proximal gracilis muscle using a novel muscle biopsy clamp^26^ that has been validated against intraoperative laser diffraction-measured sarcomere length in rabbit muscle. Harvested muscle is located proximally to the skin paddle. Using a minimal touch technique, a 1 mm thick portion of muscle is dissected for a length of 2 cm, placed in the muscle biopsy clamp, divided, and immediately placed in buffered Formalin. This length of dissection ensures that the muscle specimen is not stretched during placement of the clamps. The specimen remains in the buffered solution for 2 days. After fixation, it is removed from the buffered solution and stored in 200 ml of 1X PBS until ready for sarcomere length measurement.

### Measurement of muscle weight and videography of muscle

When the upper extremity is ready to receive the gracilis muscle, the gracilis is harvested with a goal of being revascularized within 60 min. During this time, the gracilis is weighed, and its dimensions are documented using high resolution pictures and video holding the distal tendon up and rotating the muscle next to a ruler. Images of the muscle are used to measure muscle and tendon length while the video is used to obtain volumetric measurements.

## Procedure

### Setup of USB data acquisition device (DAQ)

1. Connect the USB DAQ to the computer with LabVIEW installed. Connect the strain gauge amplifier to the DAQ.
2. Load custom data acquisition program.
3. Confirm all devices are working.

### Surgical technique for preparing the gracilis MTU

**Figure 8:**
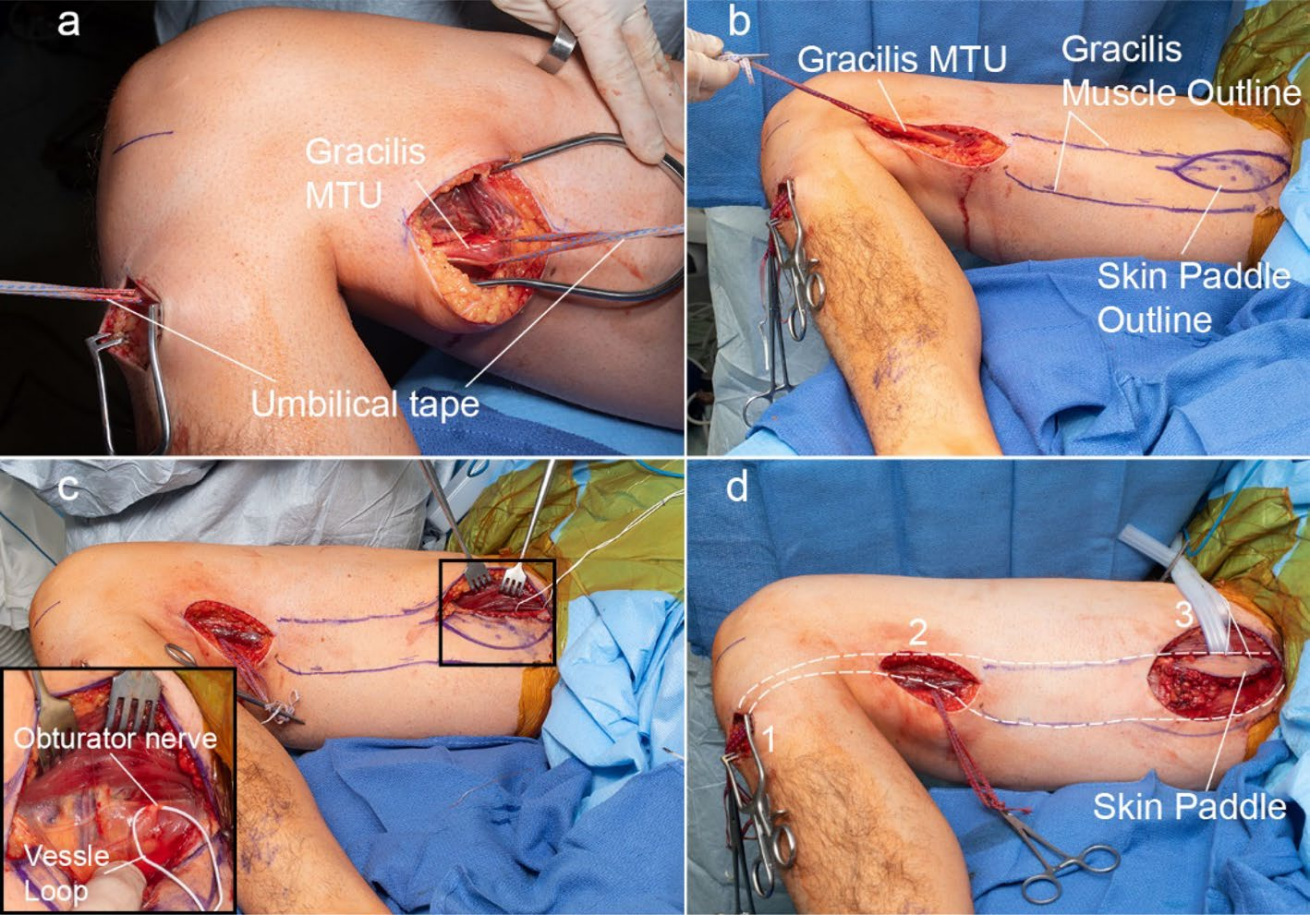
Surgical procedure for preparing the gracilis MTU for testing. (**a**) The gracilis tendon is freed from surrounding soft tissue between the tibia incision and distal medial thigh incision. Umbilical tape is place around the gracilis MTU at incision 1 and incision 2. Soft tissue attachments to the tendon are dissected until the tendon glides freely when either tether is pulled. (**b**) The outline of the gracilis and skin paddle is drawn on the medial aspect of the thigh. (**c**) The obturator motor branch is located and identified using a vessel loop. (**d**) The gracilis MTU ready for intraoperative testing after the proximal paddle incision is made and proximal gracilis tendon identified. (1) Tibia incision, (2) distal medial thigh incision, (3) proximal medial thigh incision.

4. The patient is placed in a supine position and undergoes a general endotracheal anesthesia after which paralytic agents are restricted secondary to the need for evaluation of donor motor nerves for the brachial plexus reconstruction. The patient is positioned in a slight beach chair position and the entire lower extremity is prepped and draped.
5. The gracilis harvest commences with the identification of the distal gracilis tendon at the level of the *pes anserine* of the medial proximal tibia. A longitudinal incision is made over the *pes anserine*, the sartorius fascia elevated and the gracilis and semitendinosus tendons are identified. Umbilical tape is placed around the gracilis tendon and the soft tissue attachments are methodically dissected off the tendon proximally while preserving the gracilis insertion. A longitudinal distal medial thigh incision is made to identify the muscle tendon junction of the gracilis. The gracilis tendon is freed from all surrounding soft tissue between both the tibia and distal thigh incisions. This was verified by ensuring the gracilis tendon glides freely between both incisions (Fig. 8a).
6. The gracilis tendon at the distal thigh incision is pulled using the umbilical tape and the outline of the gracilis drawn on the proximal thigh (Fig. 8b). A handheld Doppler is used to define the anterior and posterior borders of the gracilis muscle as well as the proximal vascular perforators to the skin paddle. The skin paddle is then drawn about the vascular perforators (Fig. 8b), which is approximately 6–10 cm from the groin crease.
7. An incision is made along the anterior skin paddle outline and dissection was carried through the adductor longus fascia. The adductor longus fascia is sewn to the anterior skin paddle to prevent shearing of the skin paddle.
8. The vascular pedicle and the obturator motor branch to the gracilis are identified between the adductor longus and the gracilis. The nerve is verified using a handheld nerve stimulator and a vessel loop is placed around the obturator motor branch to the gracilis (Fig. 8c). The vascular perforators to the adductor longus are ligated and the vascular pedicle (artery and two venae commitantes) are dissected to the level of the profunda femoral vessels.
9. The obturator motor branch to the gracilis is carefully dissected as far proximally as possible. The proximal gracilis tendon is visualized and cleared so its insertion on inferior pubic ramus is visible. The incision on the posterior skin paddle outline is made and dissection was done such that a fascial sleeve was maintained around the gracilis muscle (Fig. 8d).

### Intraoperative measurement.

#### Measurement of MTU length

**Figure 9:**
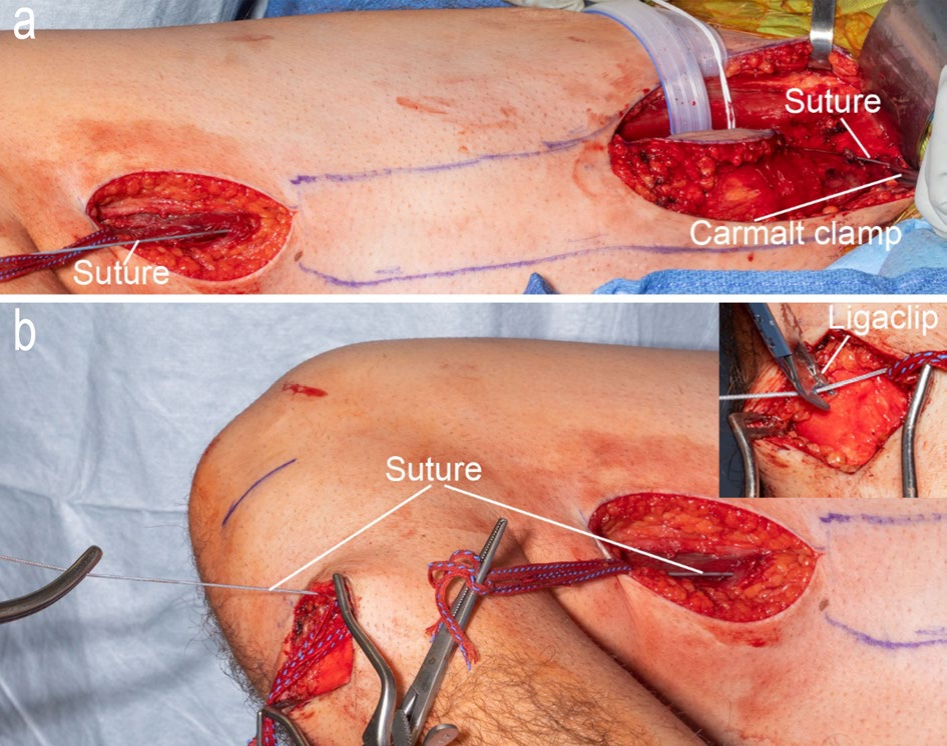
MTU length measurement. (**a**) One end of the suture is held at the gracilis origin and threaded from the proximal thigh incision to the distal thigh incision. (**b**) The suture is passed from the distal thigh incision out through the incision at the *pes anserine*. The inset image shows a titanium Ligaclip being used to mark the length of the MTU at the *pes anserine*.

10. A number 2 fiber wire suture (Arthrex, Naples FL) is passed from the proximal thigh incision, following the anterior edge of the gracilis, to the distal thigh incision, and then out the incision over the *pes anerine*. A Carmalt clamp holds the end of the fiber wire suture onto the pubic ramus origin of the gracilis muscle (Fig. 9a).
11. The hip and knee are placed in the specific joint configuration (JC) orientations described in Fig. 7 starting with the shortest length first (JC1), the distal fiber wire suture is pulled taught and marked with a Ligaclip at the level of the tibial tubercle (inset Fig. 9b).
12. The leg is manipulated to the other joint configurations, each time marking the length of the MTU on the suture. The fiber wire suture is removed, a knot is tied on the distal end and is passed off the surgical field where the measurements are subsequently determined in the laboratory.

#### Measurement of muscle force with intraoperative stimulation of the motor nerve

**Figure 10:**
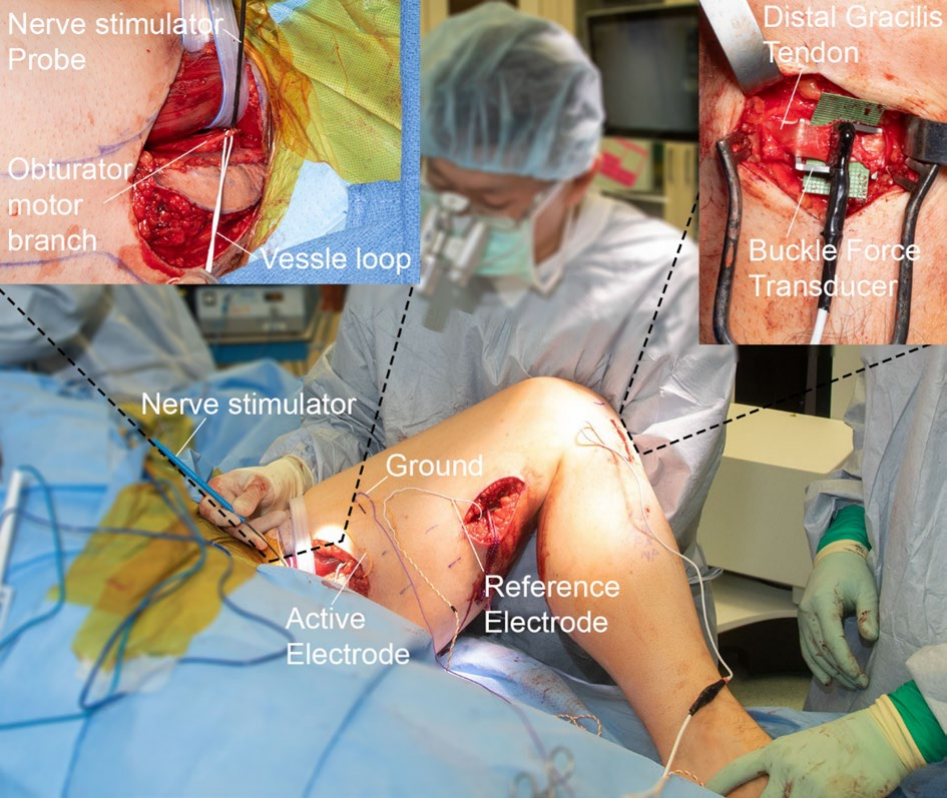
Intraoperative muscle stimulation and force measurement. Image illustrates the electrode placement required when stimulating the muscle. Left inset image shows the obturator nerve on hook of the nerve stimulator. Right inset image shows the distal tendon woven through the BFT arms. After the BFT is mounted, the incision is temporarily closed with surgical staples and the trailing wire is secured along the leg.

13. Start custom data capture code.
14. The surgeon mounts the BFT on the distal gracilis tendon at the *pes anserine*. To prevent shifting or unwanted movement of the transducer, it is placed under the skin, and the skin temporarily closed with surgical staples (Ethicon, Johnson and Johnson, Cincinnati, OH). The transducer cord is stapled along the tibia in a “S” configuration to prevent traction displacement.
15. The BFT cable is dangled off the surgical table towards the research scientist, who connects the cable to the strain gauge amplifier input.
16. The nerve stimulator probe is bent with a gentle hook and the obturator motor nerve branch to the gracilis muscle is placed on the hook approximately 3–4 cm proximal to its entrance into the gracilis muscle. A test stimulation is performed to verify muscle contraction as well as force transducer function. Care is taken by the surgeon not to put undue traction on the obturator nerve.
17. The active electrode is placed near the anticipated motor end plate, the reference electrode is placed distal in subcutaneous tissue near the gracilis tendon, and the ground is place between the active electrode and stimulator (Fig. 10).
18. The system is checked with several test stimulations prior to commencement of testing. Refer to troubleshooting table (Table 1).
19. The leg is moved to the first joint configuration and held in place by the surgeon and assistant.
20. A maximal compound muscle action potential (CMAP) response is obtained by stimulating the obturator nerve with square-wave pulses of 0.1 ms duration and intensity starting at 1 mA. The current at which a maximal CMAP response is obtained is noted and reduced by 50%, ensuring submaximal stimulation.
21. The muscle is stimulated for two consecutive trials at 2 Hz each lasting two seconds. After a rest period of 10 s, the muscle is then stimulated for two consecutive trials at 20 Hz each lasting two seconds. Refer to troubleshooting table (Table 1).
22. The stimulation current is reduced to 25% of the current required to obtain a maximal CMAP and the 2 Hz and 20 Hz trials are repeated. Refer to troubleshooting table (Table 1).
23. Steps 20–22 are repeated for each joint configuration. These nine stimulation trials are captured for each joint configuration spanning the muscle excursion range (JC1 to JC4).
24. Once all testing is completed for each joint configuration, the force transducer and EMG electrodes are removed.

**Table 1:** Troubleshooting table.

Step	Problem	Possible reason	Solution
18	No signal from BFT	Disconnected cable	Confirm good connection from BFT to strain gauge amplifier
		Faulty BFT	Ask the surgeon to remove the BFT from the patient's leg and gently tap the transducer. The BFT is sensitive to pick up on small forces. Observe if there are any changes in its signal. If there is no response, switch to the backup BFT
	No response observed	Stimulator not on obturator nerve	Ask the surgeon to confirm if the stimulator electrode is on the correct nerve
21	BFT signal noisy	Electrocautery induced interference	Confirm there is no electrical interference
		Connection from strain gauge amplifier to USB DAQ slack	Confirm there is a tight connection
		MTU not freed from surrounding tissue or BFT caught on surround tissue	Ask the surgeon to confirm the BFT placement on the tendon
	BFT signal clipping	Gain value too low	Increase gain on strain gauge amplifier
22	No response observed	25% of CMAP current too low to cause muscle contraction	Ask the neurophysiologist to increase the stimulation current

#### Muscle biopsy

**Figure 11:**
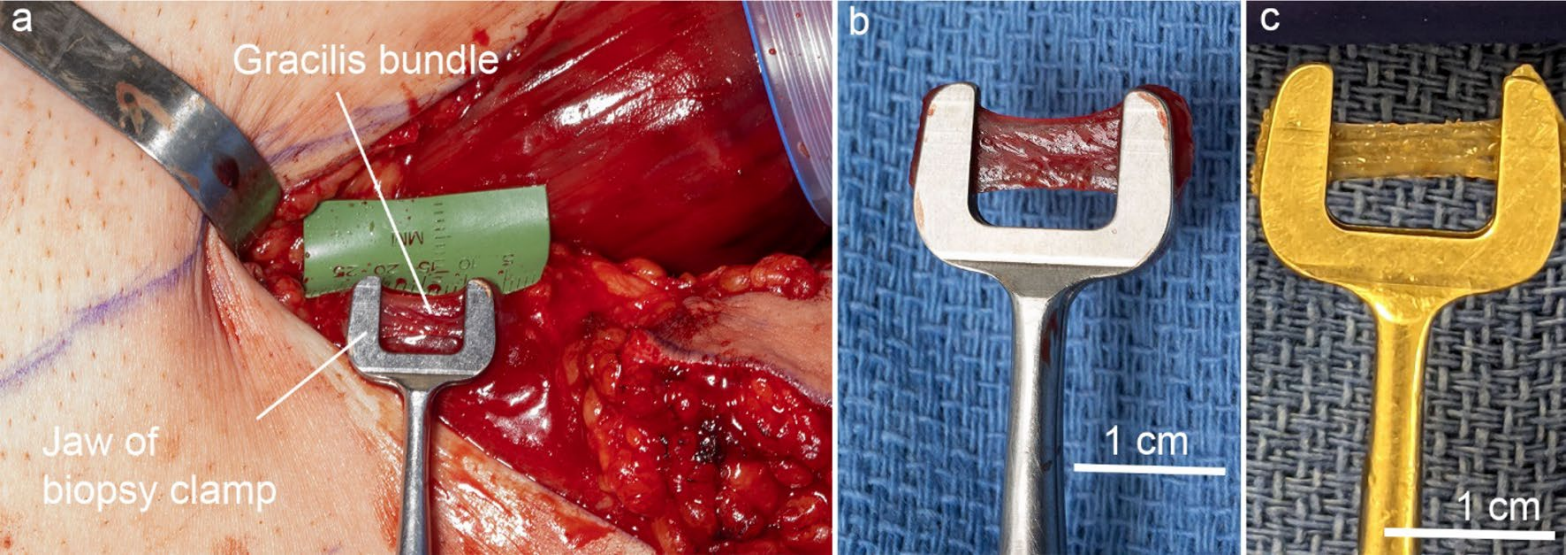
Muscle biopsy. (**a**) The gracilis bundle is positioned within the biopsy clamp and the clamp is engaged. (**b**) Biopsy sample after release from surround tissue and dissection from the gracilis. (**c**) Biopsy sample after fixation.

25. The leg is positioned to the shortest muscle length, JC1.
26. The surgeon positions the biopsy clamp and it is engaged after ensuring the muscle is in its resting position for that joint configuration (Fig. 11a) and remains unstretched.
27. The sample is bluntly dissected and freed from surrounding tissue so that when it is placed in the clamp jaws the muscle bundle is not stretched (Fig. 11b).
28. The isolated muscle fiber bundle is released proximally and distally to the clamp.
29. Immediately after the biopsy is taken, the surgeon places the clamp into a 50 mL conical tube containing 10% buffered Formalin that is held by the research scientist located on the periphery of the surgical field. The research scientist sets aside the submerged sample and provides the surgeon with another conical tube containing Formalin in preparation for the next biopsy sample.
30. Steps 26–27 are repeated for the remaining joint configurations.
31. After all the biopsy samples are obtained for each joint configuration, the research scientist covers each conical tube with parafilm, places the lid on and stores them in a biohazard transportation container. The biopsy samples will remain in Formalin for 2 days after which they are removed (Fig. 11c) and stored in 200 ml of 1X PBS solution.
32. A tray table covered with sterile plastic is prepared to be used when weighing the muscle and capturing muscle images.

#### Muscle weight and videography

**Figure 12:**
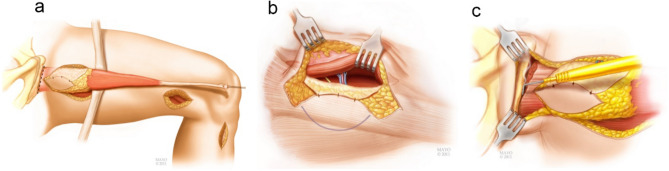
Gracilis Harvest. (**a**) Release of gracilis distally and passed through proximal thigh incision. (**b**) Gracilis neurovascular bundle. (**c**) release of gracilis proximally. (Printed with permission of the Mayo Foundation for Medical Education and Research. All rights reserved, copyright 2010).

33. Gracilis harvest continues by division of the obturator motor nerve as proximal as possible and detachment of the distal tendon at the tibia.
34. The gracilis muscle is delivered into the proximal thigh wound (Fig. 12a) and the fascia of the adductor magnus is elevated so that a fascial sling surrounds the proximal gracilis. The proximal attachment to the pubic rami is taken down (Fig. 12c). At this time the gracilis is completely free with the exception of its vascular pedicle (Fig. 12b). The muscle is temporarily left in its native bed by placing skin staples on the skin paddle to the anterior skin edge until the muscle is ready to be transferred.
35. When the upper extremity is ready to receive the gracilis muscle, the vascular pedicle of the gracilis is visualized and the artery and two venae commitantes are ligated and the ischemic clock is started.
36. The gracilis muscle is placed in a sterile bowl and weighed.
37. The surgeon holds the gracilis MTU at the distal tendon allowing the muscle to rotate along its long axis next to a ruler. A video of the rotating muscle is recorded.
38. The freed muscle is placed on a sterile surgical towel and photographed next to a ruler which provided calibration.
39. Once this is complete, the gracilis is transferred to the upper extremity and the remainder of the reconstruction is completed.

## Troubleshooting (Table 1)

## Timing

These times are estimates for a surgeon and research scientist with experience performing this protocol. When attempting this protocol for the first time, aspects of the protocol may take longer, particularly the muscle stimulation and force measurement steps.

Steps 1–2, setup of BFT connected devices: 10–15 min.

Steps 3–8, preparing gracilis muscle for harvest: 1.5–2 h.

Steps 9–11, MTU length measurement: 5–10 min.

Steps 12–23, muscle force measurement: 25–35 min.

Steps 24–29, muscle biopsy samples: 5 min.

Steps 30–35, muscle weighing and photography: 5 min.

## Anticipated results

**Figure 13:**
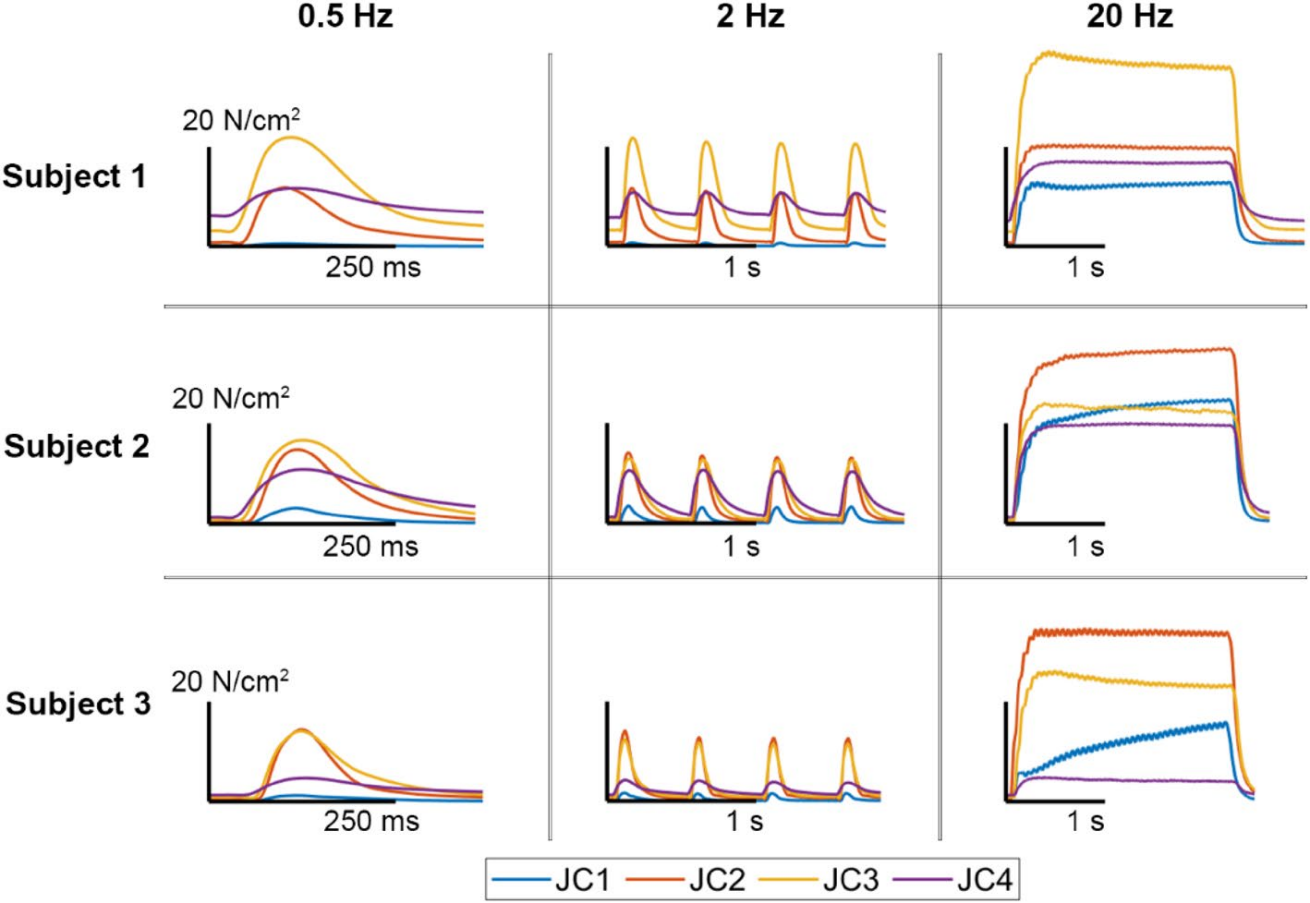
Intraoperative contractile record for three subjects. The columns show the CMAP, 2 Hz and 20 Hz muscle response for each joint configuration (JC). Data were filtered using a fourth-order low pass Butterworth filter and a cutoff frequency of 30 Hz. Total force was normalized to muscle physiological cross-sectional area. Time scale bars are labeled for each plot. Hip abduction (HA) and knee flexion (KF) angles for each JC are written as (HA, KF). JC1 (45°, 130°), JC2 (45°, 90°), JC3 (45°, 45°) and JC4 (60°, 0°). Hip flexion was constant at 60° throughout. The baseline force for each contractile record shows the amount of passive tension present in the gracilis MTU. There is an increase passive tension from JC1 to JC4.

Buckle force data for three subjects are shown in Fig. 13. Maximal CMAP twitch, 2 Hz and 20 Hz stimulation trials for each joint configuration are presented in each column. Unfused tetanic contractions are observed at 20 Hz for each joint configuration. To evaluate intra-subject variability, peak force was compared between the two 2 Hz and 20 Hz trials. The root mean squared error was calculated to be 2.4 N and 9.8 N for the 2 Hz and 20 Hz trials, respectively.

**Figure 14:**
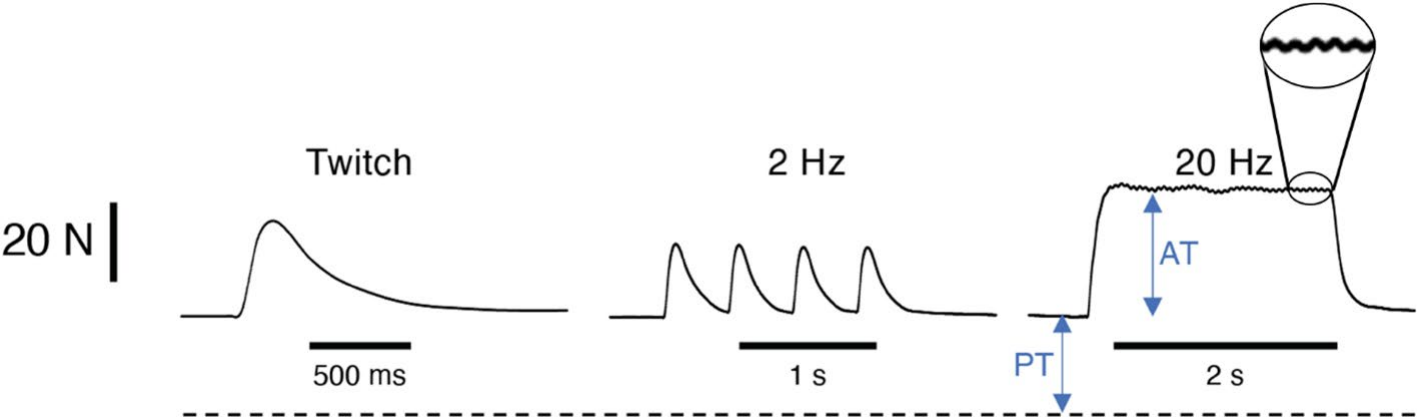
Intraoperative contractile record from BFT of gracilis muscle. Active tension (AT) and Passive tension (PT) are indicated in the figure. The dotted line represents zero tension, so this muscle is under about 20 N of passive tension in vivo. Inset represents the plateau of the 20 Hz stimulation showing a 20 Hz ripple.

A more detailed look at the force traces is presented in Fig. 14. Note that the twitch demonstrates the typical asymmetrical rise time and lengthened fall time characteristic of skeletal muscle (see comparisons in Fig. 1A of Oda, et al.^28^ and Fig. 1 of Lieber^29^) indicating that it is free to develop force and that it is sufficiently activated by the motor nerve to recruit most or all of the motor units. Note also that at 2 Hz stimulation, the twitch is nearly completely relaxed while at 20 Hz stimulation, temporal summation of contractile events occurs leading to tetanic contraction as the active tension (AT). The high frequency 20 Hz tension ripple can be seen at the peak of the tetanic contraction (Fig. 14, inset).

**Figure 15:**
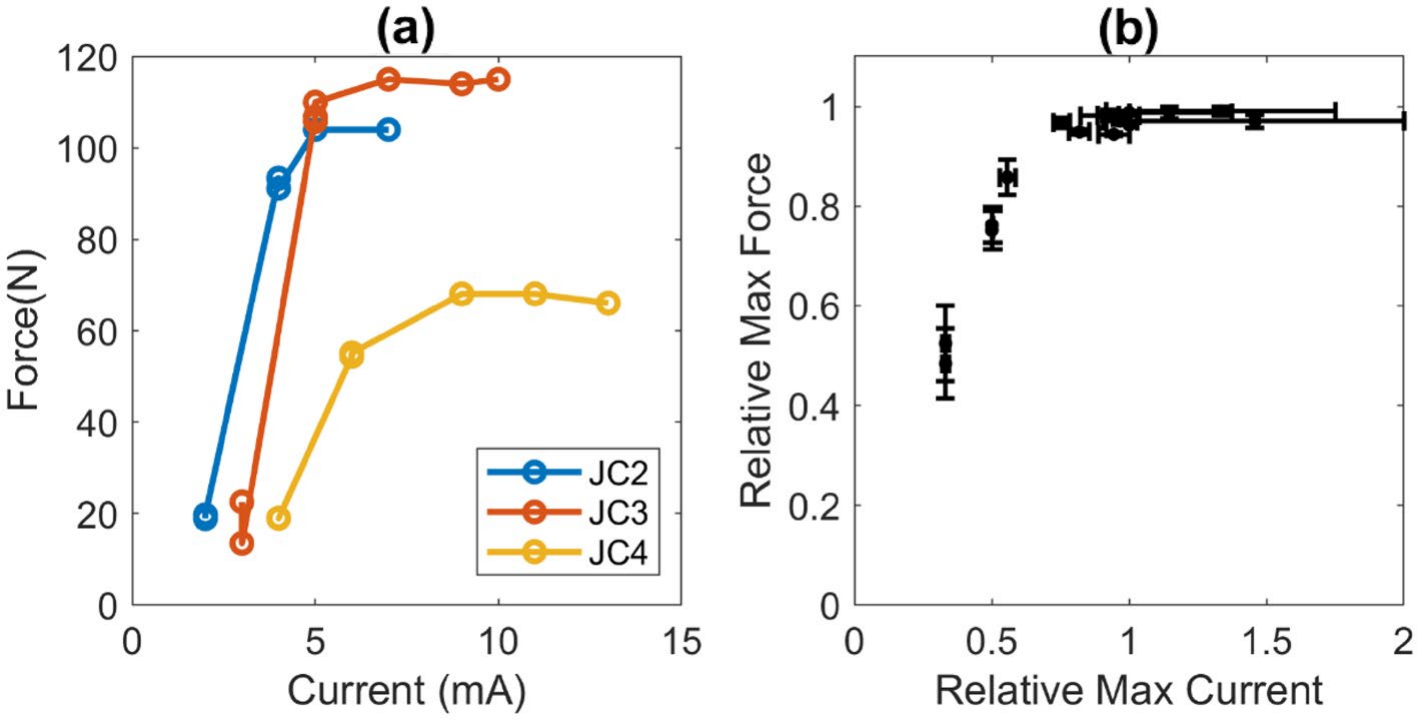
Recruitment curve. (**a**) Twitch force versus stimulation current for one subject. (**b**) Recruitment curved calculated for 12 subjects. Active force and stimulation current were normalized to their respective maximum value at each joint configuration during the CMAP probing trials described by step 20 above.

The variation in muscle force with stimulation intensity is depicted in Fig. 15. Figure 15a shows the raw experimental values for one subject where the force increases with increasing stimulation current until it plateaus. Figure 15b shows the normalized force-current relationship averaged over 12 subjects. These data were recorded during step 20 when the neurophysiologist gradually increases the stimulation current until a maximal CMAP response is recorded.

**Figure 16:**
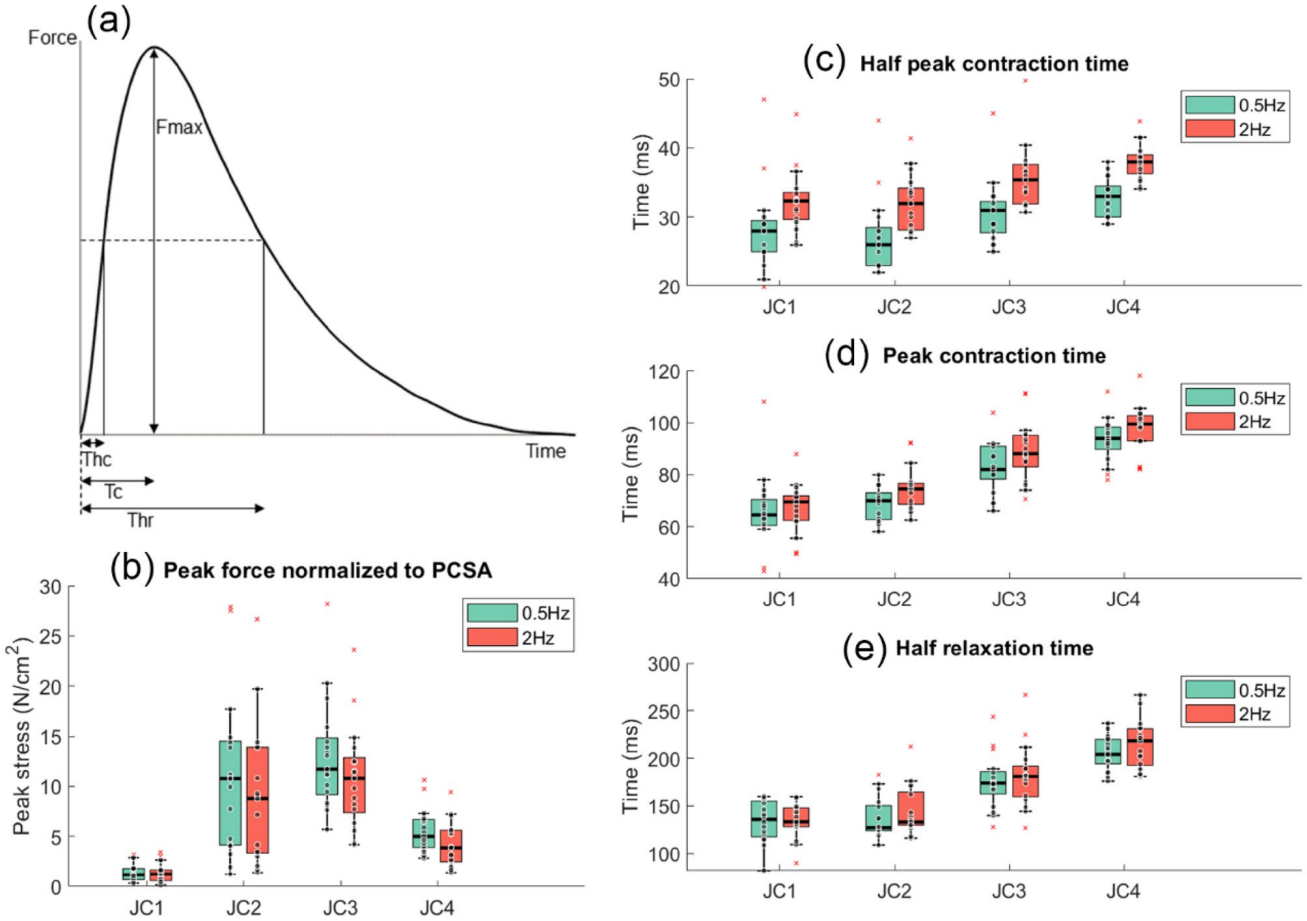
Muscle contractile parameters for 0.5 Hz and 2 Hz activation. (**a**) Definition of the twitch contractile parameters; *Thc* time from start of contraction to moment when force is half its maximum value, *Tc* time from start of contraction to moment when force is at its maximum value, *Thr* time from start of contraction to when force decreases to half its maximum value during the relaxation phase, *Fmax* maximum twitch force. (**b**) Peak force normalized to muscle physiological cross-sectional area. (**c**) Half peak contraction time (Thc). (**d**) Peak contraction time (Tc). (**e**) Half relaxation time (Thr). Twitch parameters for 0.5 Hz are compared to the first 2 Hz contraction for seventeen subjects (n = 17).

Twitch contractile parameters and their definitions are shown in Fig. 16. Normalized peak force of the first 2 Hz twitch and 0.5 Hz twitch for seventeen subjects were compared. The ratio of peak stress was approximately 0.8 and indicate that nearly all the motor units are being activated during the submaximal stimulated 2 Hz trials. The relatively long contraction and relaxation times (Fig. 16c–e) support the fact that the gracilis is comprised of about 75% slow muscle fibers^30^.

**Figure 17:**
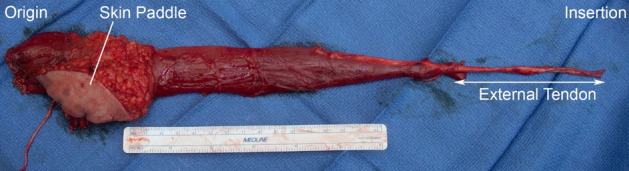
Harvested gracilis MTU. The harvested gracilis is placed on a sterile towel and photographed next to a sterile ruler. Muscle and tendon length are measured from these images.

The harvested MTU is shown in Fig. 17. MTU, muscle and external tendon slack length are measured from these images. External tendon length is measured from the point where the muscle fibers terminate distally. The skin paddle is shown near the muscle origin along with neurovascular supply and adipose tissue.

## Data Availability

Raw data obtained using this protocol are available from the corresponding author upon request.

## Code availability

Custom LabVIEW code used in the protocol will be made available upon request to the corresponding author.
